# Radar Detection of Fluctuating Targets under Heavy-Tailed Clutter Using Track-Before-Detect

**DOI:** 10.3390/s18072241

**Published:** 2018-07-12

**Authors:** Jie Gao, Jinsong Du, Wei Wang

**Affiliations:** 1 Shenyang Institute of Automation, Chinese Academy of Sciences, Shenyang 110016, China; jsdu@sia.cn (J.D.); wangwei2@sia.cn (W.W.); 2 University of Chinese Academy of Sciences, Beijing 100049, China

**Keywords:** target detection, radar systems, K-distributed clutter, heavy-tailed, Swerling target, track-before-detect (TBD)

## Abstract

This paper considers the detection of fluctuating targets in heavy-tailed clutter through the use of dynamic programming based on track-before-detect (DP–TBD) in radar systems. The clutter is modeled in terms of K-distribution, which can be widely used to describe non-Gaussian clutter received from high-resolution radars and radars working at small grazing angles. Swerling type 1 is considered to describe the target fluctuation between scans. Conventional TBD techniques suffer from significant performance loss in heavy-tailed environments due to the more frequent occurrences of target-like outliers. In this paper, we resort to a DP–TBD algorithm based on prior information, which can enhance the detection performance by using the environment and target fluctuating information during the integration process of TBD. Under non-Gaussian background, the expressions of the likelihood ratio merit function for Swerling type 1 targets are derived first. However, the closed-form of the merit function is difficult to obtain. In order to reduce the complexity of evaluating the merit function and the computational load, an efficient approximation method as well as a two-stage detection approach is proposed and used in the integration process. Finally, several numerical simulations of the new strategy and the comparisons are presented to verify that the proposed algorithm can improve the detection performance, especially for fluctuating targets in heavy-tailed clutter.

## 1. Introduction

The detection of fluctuating targets with low signal-to-clutter ratio (SCR) is of significant importance in radar systems. Conventional detecting and tracking algorithms use thresholded detection as input. A target with low signal-to-clutter ratio is often lost due to information being irreversibly discarded after thresholding. Multi-frame integration is an effective strategy used in radar applications to detect dim targets by integrating signal returns over multiple consecutive scans. In the presence of a moving target, multi-frame integration requires track-before-detect (TBD) techniques to correctly correlate data over time.

Dynamic programming based on TBD (DP–TBD) is one of the TBD techniques [1,2], that has attracted extensive attention for the advantages of simplicity and needing less information. It transforms the integration into an optimal estimation of the physically admissible trajectory by maximal integration value of the merit function, which is a kind of multi-frame test statistic. DP–TBD can detect a target of arbitrary motion form and has been widely applied to several kinds of sensors [3,4]. In order to solve the problem of high-dimensional maximization under a multi-target environment, a novel partition method to cluster targets into well separated groups was proposed in [5]. In [6,7], the track formation procedure with successive track cancellation (STC) was described to overcome the performance loss when targets are closely spaced. Meanwhile, research on the merit function of DP–TBD has also been widely carried out in recent years. In [8,9], the expressions for the log-likelihood ratio (LLR), which can better discriminate clutter-plus-target measurements from clutter only measurements, were derived and used. In addition, a low-complexity power-efficient TBD procedure, where the generalized likelihood ratio test (GLRT) [10,11,12] was solved using a Viterbi-like tracking algorithm, was proposed in [13]. To reduce the big computational burden of DP–TBD, computationally efficient DP–TBD algorithms were derived in [14,15] respectively.

The above quoted papers on DP–TBD techniques always assumed that the background is Gaussian distributed with known power. However, for high-resolution radars and radars at small grazing angle, the Gaussian assumption may not be adequate. In this case, more heavy-tailed background models should be considered in the real world. Weibull distribution, log-normal distribution and K-distribution are the commonly compound-Gaussian background models used in radar communities. This paper is mainly concerned with K-distribution, which is widely used in high-resolution radar detection systems. K-distribution [16,17] was derived from a paper by Eric Jakeman and Peter Pusey (1978) who used it to model microwave sea echo. It has been found to be a suitable model for heavy-tailed background in radar systems [18], since it provides an excellent agreement between theoretical and experimental data. K-distribution also arised as the consequence of a statistical or probabilistic model used in synthetic aperture radar (SAR) imagery.

As the signal strength may change from scan to scan, these fluctuations should be taken into account when building the measurement-based model. A Swerling family of target amplitude fluctuation models is commonly used to capture the radar-cross section (RCS) changes over time [19]. Swerling targets of type 0 can be used to model a target with constant RCS, while Swerling targets of type 1 is used to model a target whose RCS fluctuates according to the exponential density in radar systems.

Target detection in K-distributed background is more challenging than in Gaussian or Rayleigh distributed background due to the higher likelihood of target-like outliers, especially for fluctuating targets. Besides, it is inefficient and computationally costly to carry out an accurate search for all the discrete states, as the surveillance region is much larger than the size of a target, such as radar target detection. In this paper, attention is devoted to the detection of a Swerling target of type 1 in a surveillance region characterized by K-distributed background through the use of DP–TBD. Moreover, by employing a two-stage detection approach, the proposed algorithm is able to achieve further computational reduction. The main contributions of this paper are given as below:

1. In order to limit complexity while still retaining the benefits of DP–TBD, we resort to a two-stage detection process with different resolution cells.
2. For typical non-Gaussian distributed clutter (K-distribution) and a typical target amplitude fluctuation model (Swerling 1), the DP–TBD algorithm based on prior information is proposed. By using the likelihood ratio merit function in DP integration, the performance loss produced by the “heavy-tailed” clutter measurements can be reduced.
3. An efficient but accurate approximation method is proposed to reduce the complexity of evaluating the merit function.

The remainder of this paper is organized as follows: Section 2 presents the notations and system models. In Section 3, a two-stage detection approach is proposed at first, and the expressions of the likelihood ratio merit function are derived in K-distributed clutter background for Swerling target of type 1; the implementation issues of the merit function are also discussed. Simulation results are showed by comparing different DP–TBD strategies in Section 4 and Section 5 provides some conclusions.

Mathematical notations used in this paper are described as follows. $s_{n}$ is the target kinematic state at scan n; $A_{n}$ denotes an amplitude from the Swerling 1 target and $c_{n}\left(i,j\right)$ denotes the K-distributed clutter; $a_{n}$ is the measurement amplitude in K-distributed clutter background. $I\left(s_{n}\right)$ is defined as the merit function at scan n; $V\left(s_{n-1}\right)$ is defined as the maximal integration value of all the admissible trajectories; $\tau \left(s_{n}\right)$ is the collection of states at scan n for which transition to $s_{n}$ is possible; $\Psi \left(s_{n}\right)$ is the retracing function, indicating the best state of the previous scan.

## 2. Models and Notations

### 2.1. Kinematic Model

**Figure 1:**
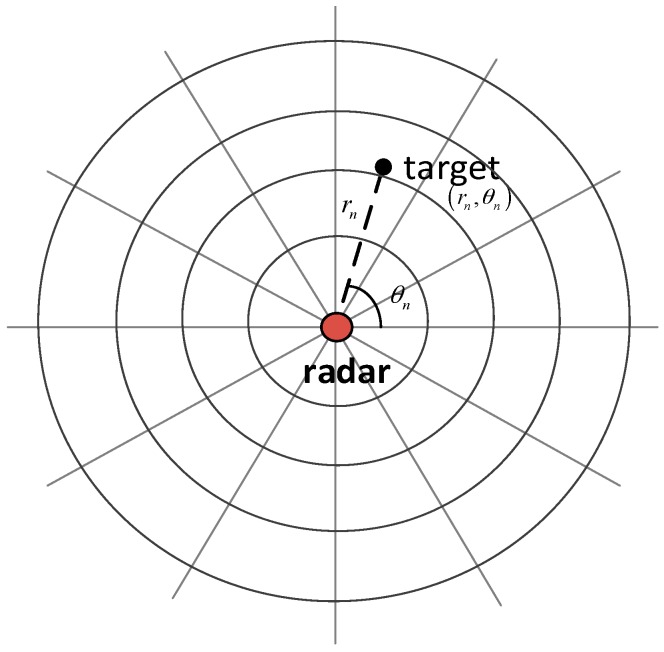
Radar surveillance region illustration.

As shown in Figure 1, we assume that there is only one target in the surveillance region, whose kinematic state at scan n is denoted by the vector $s_{n}$. The kinematic vector $s_{n}$ is specified by:

(1) $$s_{n}={\left[r_{n},\theta_{n}\right]}^{\prime }\in R^{2},\;1\leq n\leq N$$

where ′ denotes matrix transpose, $r_{n}$ and $\theta_{n}$ denote the range and azimuth measurement, respectively, $R^{2}$ denotes the two-dimensional state space, and N denotes the number of consecutive frames processed in a DP–TBD integration batch. The evolution of the target state is modeled by the linear process as:

(2) $$s_{n}=Fs_{n-1}+w_{n}$$

The term $w_{n}$ is the process noise, F is the transition matrix.

Every real target must comply with some physical constraints on its kinematics, such as the maximum target velocity considered in this paper. The radial and tangential velocity can be calculated by two successive scans, which are given by:

(3) $$\begin{array}{l} v_{n-radial}=\frac{r_{n}-[r_{n-1}\times \cos\left(\theta_{n}-\theta_{n-1}\right)]}{T}\\ v_{n-\tan gential}=\frac{r_{n-1}\times \sin\left(\theta_{n}-\theta_{n-1}\right)}{T} \end{array}$$

where T denotes the time interval between successive scans.

### 2.2. Measurement-Based Model

The measurement data consists of $M_{r}$ cells in the range-dimension and $M_{\theta }$ cells in the azimuth-dimension. If no target exists (hypothesis $H_{0}$), the (i,j)th recorded resolution cell, $z_{n}\left(i,j\right)$ $1\leq i\leq M_{r},1\leq j\leq M_{\theta }$, at scan n can be expressed as [20]:

(4) $$z_{n}\left(i,j\right)=c_{n}\left(i,j\right)$$

while in the presence of a target (hypothesis $H_{1}$), the recorded resolution cell $z_{n}\left(i,j\right)$ can be expressed as:

(5) $$z_{n}\left(i,j\right)=A_{n}+c_{n}\left(i,j\right)$$

where $A_{n}$ denotes a complex fluctuated amplitude measurement from the target, and $c_{n}\left(i,j\right)$ denotes the K-distributed clutter, which is assumed in this paper.

A Swerling 1 fluctuation model supposes that returned signal power per pulse is constant during a single scan, but fluctuates independently from scan to scan. The probability density function (PDF) of the Swerling 1 target amplitude $A_{n}$ is given by:

(6) $$p\left(A_{n}\right)=\frac{2A_{n}}{\bar{\sigma }}\exp\left(-\frac{{A_{n}}^{2}}{\bar{\sigma }}\right)$$

with $\bar{\sigma }$ being the mean squared target amplitude.

### 2.3. K-Distributed Clutter Model

The K-distributed model is proposed as a model for radar clutter in this paper, which has the probability density function as:

(7) $$p\left(a_{n}\right)=\frac{4{a_{n}}^{\alpha }}{\beta^{\left(\alpha +1\right)/2}\Gamma \left(\alpha \right)}{Κ}_{\alpha -1}\left(\frac{2a_{n}}{\beta^{1/2}}\right)$$

**Figure 2:**
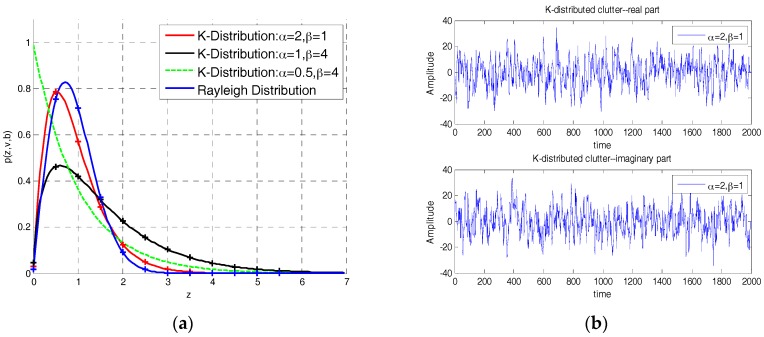
K-distribution (**a**) probability density functions (PDFs) of K and Rayleigh distribution for various shape and scale parameters; (**b**) K-distributed clutter including real part and imaginary part.

In formula, Γ(⋅) denotes the Gamma function and $K_{\alpha -1}\left(\cdot \right)$ denotes the modified Bessel function of the second kind, $a_{n}$ is the measurement amplitude, β is scale parameter which describes the intensity of the clutter, and α is the shape parameter which determines the shape of the distribution function. For α→∞, the K-distribution turns into the Rayleigh distribution. PDFs of K- and Rayleigh- distributions are shown in Figure 2a while the K-distributed clutter (α=2,β=1) is shown in Figure 2b.

Meanwhile, K-distribution can also be viewed as a Rayleigh distribution modulated by a Gamma distribution for convenience:

(8) $$p\left(a_{n}\right)=\int_{0}^{+\infty }p\left(a_{n}|\eta \right)p\left(\eta \right)d\eta$$

where

(9) $$p\left(a_{n}|\eta \right)=\frac{2a_{n}}{\eta }\exp\left(-\frac{{a_{n}}^{2}}{\eta }\right)$$

(10) $$p\left(\eta \right)=\frac{\eta^{\alpha -1}}{\beta^{\alpha }\Gamma \left(\alpha \right)}\exp\left(-\frac{\eta }{\beta }\right)$$

## 3. Development of the Proposed Strategies

The DP–TBD algorithm decomposes the integration among N successive scans into N sub-processes. The *n*th sub-process contains all the measurements up to scan n. The target can be detected and tracked by calculating the maximum of the energy integration value through a recursive model, which could be expressed as:

(11) $$V\left(s_{n}\right)=\sqrt{I\left(s_{n}\right)}+\underset{s_{n-1}\in \tau \left(s_{n-1}\right)}{\max}\left[V\left(s_{n-1}\right)\right]$$

(12) $$\Psi \left(s_{n}\right)=\arg\underset{s_{n-1}\in \tau \left(s_{n}\right)}{\max}\left[V\left(s_{n-1}\right)\right]$$

where $I\left(s_{n}\right)$ is defined as the merit function at scan n; $V\left(s_{n-1}\right)$ is defined as the maximal integration value of all the admissible trajectories; $\tau \left(s_{n}\right)$ is a collection of states at scan n for which a transition to $s_{n}$ is possible, and it can be obtained by the location and maximum velocity of the target; $\Psi \left(s_{n}\right)$ is the retracing function, indicating the best state of the previous scan, which makes the integration value reach its maximum.

In summary, DP–TBD implements the equivalent of an exhaustive search in an efficient manner by enumerating and valuing all physical admissible state sequences, finally returning the state sequences whose final maximal integration value $V\left(s_{N}\right)$ exceeds a given detection threshold γ, i.e.,:

(13) $$V\left(s_{N}\right)>\gamma$$

There are mainly two problems throughout the process. Firstly, the computational complexity of DP–TBD is unaffordable in the presence of a high-mobility target when the number of resolution elements is large. The discretization of state space is always based on the sensor’s resolution so as to make full use of the measurements and achieve possibly accurate estimates. In this situation, strategies hardly lead to real-time implementable schemes, even resorting to a dynamic programming algorithm. In order to reduce the burden of computation, a two-stage detection approach is proposed in this work. Secondly, most of the previous work on DP–TBD assumed that the background model would be Rayleigh or Gaussian distribution with a known power. Such assumptions may not be adequate, as in the real world a more heavy-tailed background model than expected is often encountered. To improve the detection performance, we propose a novel DP–TBD algorithm based on the prior information to solve the aforementioned problem. In this paper, the merit function is set to be the likelihood ratio under both target-present hypothesis and null-target hypothesis in a surveillance region which is characterized by K-distributed background, and the simulated data would be tested for presenting the performance.

### 3.1. Two-Stage Detection Approach

**Figure 3:**
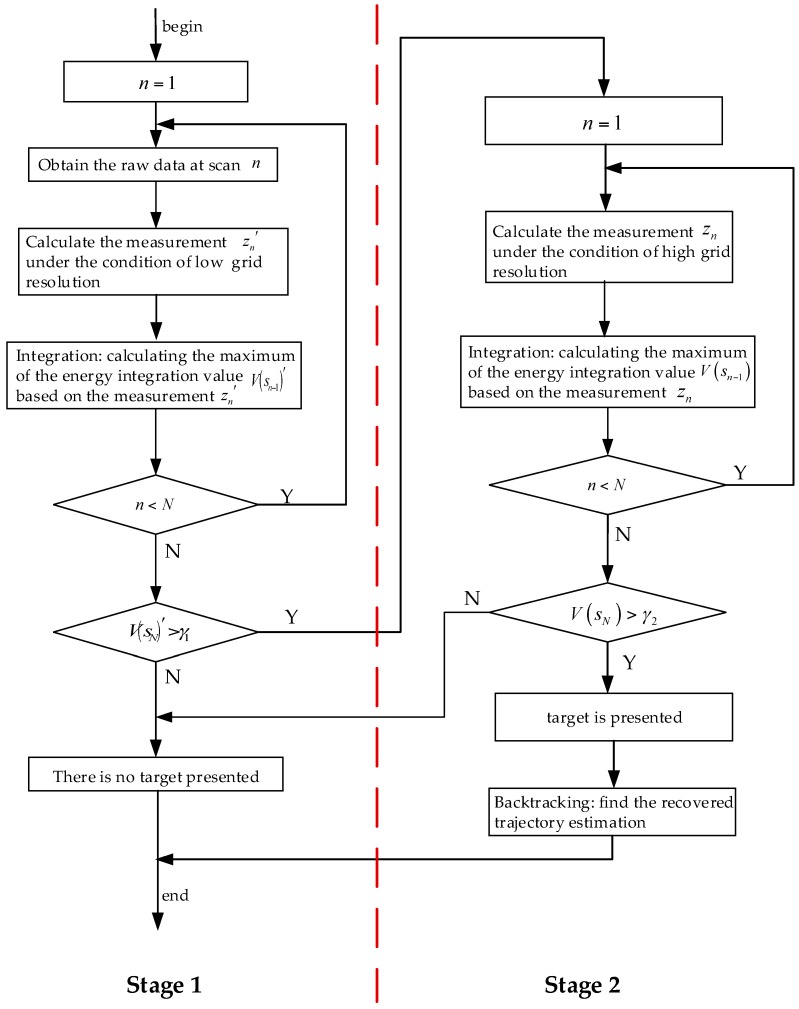
The flowchart of the two-stage detection approach.

Generally, DP–TBD is a grid-based method that estimates target trajectory by means of searching all the admissible paths in a discrete state space and the discretization of state space is based on the sensor’s resolution. It is inefficient and computationally costly to carry out an accurate search (i.e., the search grid is exactly divided based on the sensor’s resolution) since only a fraction of measurements are related to the actual target when the surveillance region is large. In order to reduce the computational load, while still retaining the benefits of TBD, here we resort to a two-stage detection approach which is illustrated in Figure 3.

At stage 1, we first obtain the raw data at scan n, and roughly calculate the measurement ${z_{n}}^{\prime }$ under the condition of low grid resolution. The target states are estimated by searching discrete grids with larger cell size based on the DP integration. After *N* times loop, the maximum of the energy integration value $V{\left(s_{N}\right)}^{\prime }$ at scan *N* could be obtained by the process. For a single target model, the maximum integration value $V{\left(s_{N}\right)}^{\prime }$ which exceeds detection threshold $\gamma_{1}$ is used to determine the existence of the target. If there is a target presented in the surveillance region, we could refine the target trajectory in stage 2.

In order to obtain a more accurate estimate, stage 2 is employed to recalculate the measurements under the high grid resolution condition. Once the maximum integration value $V\left(s_{N}\right)$ exceeds the detection threshold $\gamma_{2}$, the estimation of the final target trajectory can be obtained by backtracking. For each estimated state ${\hat{s}}_{n}$, we have:

(14) $${\hat{s}}_{n-1}=\psi \left({\hat{s}}_{n}\right),\;for\;n=N,\ldots 1$$

So the recovered trajectory estimate is ${\hat{S}}_{N}=\left\{{\hat{s}}_{1},\ldots ,{\hat{s}}_{N}\right\}$. The algorithmic description of the proposed two-stage TBD approach is shown in Table 1.

**Figure 4:**
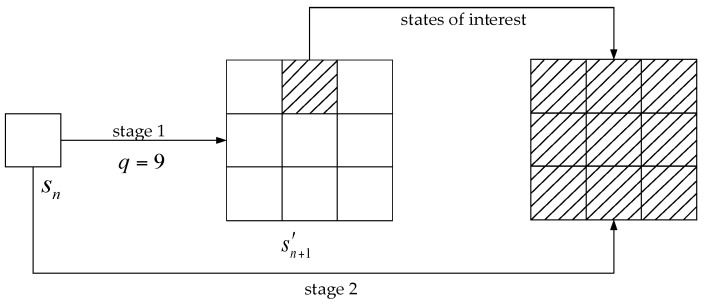
Illustration of possible transition state collection during the two-stage DP integration.

The surveillance region is divided into $M_{r}\times M_{\theta }$ grid cells based on the resolution of the radar system, i.e., ∆r and ∆θ, where $M_{r}$ and $M_{\theta }$ denote the number of cells in range and azimuth, respectively. To realize the target search with larger cell size, the state space is re-discretized by $∆r^{\prime }$ and $∆\theta^{\prime }$ to obtain $M_{r}^{\prime }\times M_{\theta }^{\prime }$ grid cells at first. As shown in Figure 4, all the measurements and DP integrations are processed in stage 1 based on the new state space, which may obtain a rough target trajectory by less computation. Then in stage 2, DP integration concentrates on the part of states which are indicated by stage 1. As calculations of less meaningful states could be avoided, the computational costs will become more reasonable.

### 3.2. Derivation and Implementation of the Merit Function

Combined (6), PDF for the Swerling 1 target in K-distributed clutter is given by:

(15) $$p\left(a_{n}|\eta ,s_{n}\right)=\frac{2a_{n}}{\eta +\bar{\sigma }}\exp\left(-\frac{{a_{n}}^{2}}{\eta +\bar{\sigma }}\right)$$

and $p\left(a_{n}|s_{n}\right)$ can be derived by marginalizing over η since η is random, i.e.,

(16) $$\begin{array}{ll} p\left(a_{n}|s_{n}\right) & =\int_{0}^{\infty }p\left(a_{n}|\eta ,s_{n}\right)p\left(\eta \right)d\eta \\ & =\int_{0}^{\infty }\frac{2a_{n}}{\eta +\bar{\sigma }}\exp\left(-\frac{{a_{n}}^{2}}{\eta +\bar{\sigma }}\right)\frac{\eta^{\alpha -1}}{\beta^{\alpha }\Gamma \left(\alpha \right)}\exp\left(-\frac{\eta }{\beta }\right)d\eta \\ & =\frac{2a_{n}}{\beta^{\alpha }\Gamma \left(\alpha \right)}\int_{0}^{\infty }\frac{\eta^{\alpha -1}}{\eta +\bar{\sigma }}\exp\left(-\frac{{a_{n}}^{2}}{\eta +\bar{\sigma }}\right)\exp\left(-\frac{\eta }{\beta }\right)d\eta \\ & =\frac{2a_{n}}{\beta^{\alpha }\Gamma \left(\alpha \right)}\int_{0}^{\infty }f(\eta )d\eta \end{array}$$

where the integrand f(η) is given by:

(17) $$f\left(\eta \right)=\frac{\eta^{\alpha -1}}{\eta +\bar{\sigma }}\exp\left(-\frac{{a_{n}}^{2}}{\eta +\bar{\sigma }}-\frac{\eta }{\beta }\right)$$

Substituting (7) and (16) into the expression of merit function $I\left(s_{n}\right)$ at scan n, $I\left(s_{n}\right)$ can be written as:

(18) $$\begin{array}{ll} I\left(s_{n}\right) & =\ln\left(\frac{p(a_{n}|s_{n})}{p\left(a_{n}\right)}\right)\\ & =\ln\left(\frac{{a_{n}}^{-\alpha +1}\beta^{\left(-\alpha +1\right)/2}\int_{0}^{\infty }f\left(\eta \right)d\eta }{K_{\alpha -1}\left(2a_{n}/\beta^{1/2}\right)}\right) \end{array}$$

Although the integrand f(η) in (17) has no closed-form solution, it can be evaluated with reasonable accuracy by using the trapezoidal rule, i.e.,

(19) $$\int_{0}^{\infty }f\left(\eta \right)=\sum_{i=1}^{N_{sa}}\frac{f\left(\eta_{i}\right)+f\left(\eta_{i+1}\right)}{2}\delta_{i}$$

where $f\left(\eta_{i}\right)$ is sample point drawn from the time interval $\delta_{i}$, $\delta_{i}$ is a sampling interval which is short enough to cover the effective support of f(η), and $N_{sa}$ denotes the number of sample points.

The sample points can be obtained by either deterministic sampling with a uniform grid or stochastic importance sampling. Since the integrand f(η) may tend quickly towards ∞ when η→0, while tending slowly towards 0 when η→∞. A reasonable approximation obtained by deterministic uniform grid sampling or stochastic importance sampling is difficult to carry out. A grid with a variable resolution method was proposed in [21] to approximate the merit function, which also leads to high computational complexity.

**Figure 5:**
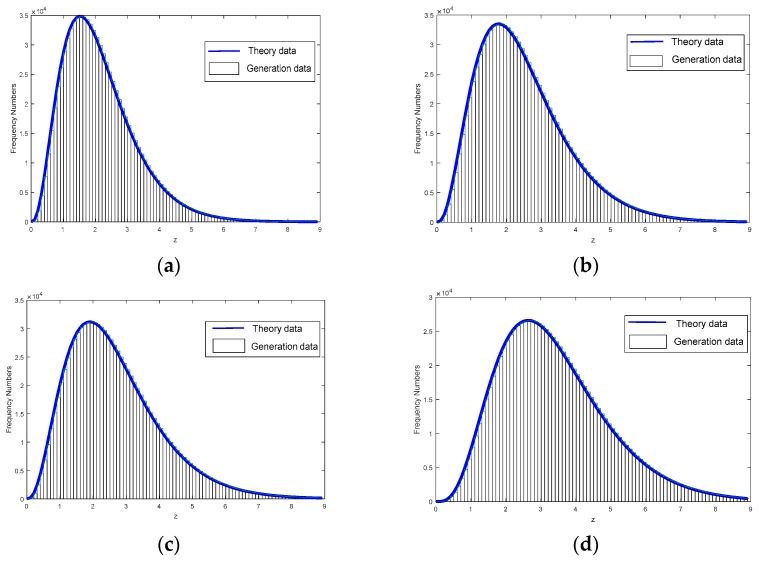
Histogram of generation data and theory PDF data with signal-to-clutter ratio (SCR) = 15 dB (**a**) α=2 and β=1; (**b**) α=3 and β=2; (**c**) α=5 and β=2; (**d**) α=10 and β=5.

In order to reduce the complexity of approximation, we could possibly circumvent these problems by generating a lookup table offline with sample points using a uniform grid. The number of sample points with uniform grid is large enough to approximate the integrand f(η) accurately. Based on the lookup table, this calculating method trades little cost of precision and memory space for a great improvement on running speed in the calculation. The histograms of generation data and theory PDF are shown in Figure 5 for the Swerling targets type 1 with different parameters. According to Figure 5, we conclude that the approximation error is negligible.

Note that the K-distribution shape parameter α and the scale parameter β are supposed to be known in the derivation of merit function. In the case where the background is significantly heavy-tailed and the parameters are unknown, we should estimate the parameters first, which can be obtained through a numerical maximization of the likelihood function. Since the maximum likelihood techniques require numerical optimization routines and evaluation of Bessel functions, they are computationally intensive and, therefore, inappropriate for evaluation of large data sets. Abraham [22] recommended moment estimators based on the first and second moments, which can be used as our estimator in this work.

## 4. Simulation

In this paper, the detection performances of conventional DP–TBD and the proposed strategy for a Swerling type 1 target are assessed. We assume that the measurement noise satisfies K-distribution, and each measurement frame consists of $M_{r}\times M_{\theta }$ = 180 × 90 resolution cells. The number of frames processed in a DP batch is N=6, while the number of possible state transitions in a scan is *q* = 9. This scenario is run 1000 times for various SCR and shape parameters while the false alarm is fixed as $P_{FA}=10^{-3}$.

### 4.1. Performance Analysis

We assess the performances of different strategies via the probability of track detection $P_{d}$, which is a performance metric for both detection and tracking performance. $P_{d}$ is defined as the probability of the maximum integration value exceeding the detection threshold, and its final position is within a certain range of the actual target position. In addition, the root-mean square error (RMSE) on the estimation of the target position is also considered, which is defined as:

(20) $$RMSE=\sqrt{E\left[e^{2}\left(s_{n}\right)|H_{1}\right]}$$

where $H_{1}$ is the event that the target is confirmed, and $e^{2}\left(s_{n}\right)$ is the Euclidean distance between the true and estimated target position.

**Figure 6:**
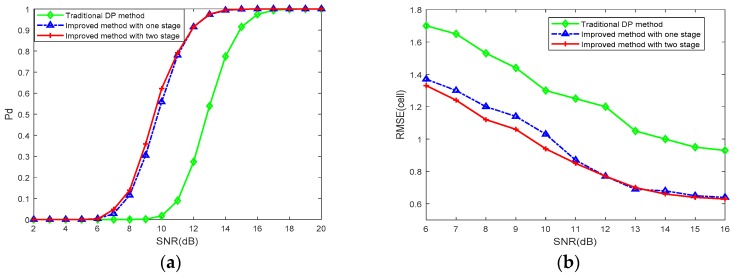
Performance and root-mean square error (RMSE) comparison of different DP–TBD integration method with α=0.5 and β=1 against signal-to-noise ratios (SNRs) from 2 dB to 20 dB. (**a**) The detection probability $P_{d}$; (**b**) the RMSE on estimated position.

Performance and RMSE comparison of the conventional DP method and the proposed method based on prior information is shown in Figure 6. For K-distributed clutter and a Swerling 1 target, the proposed method performs better than the traditional integration method. It can also be concluded that the proposed method, which is processed with only one stage (blue solid line) or two stages (red solid line), could achieve almost identical performance while the latter obtains further computational reduction.

**Figure 7:**
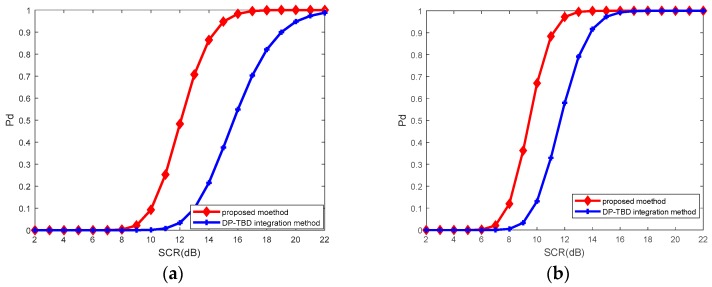

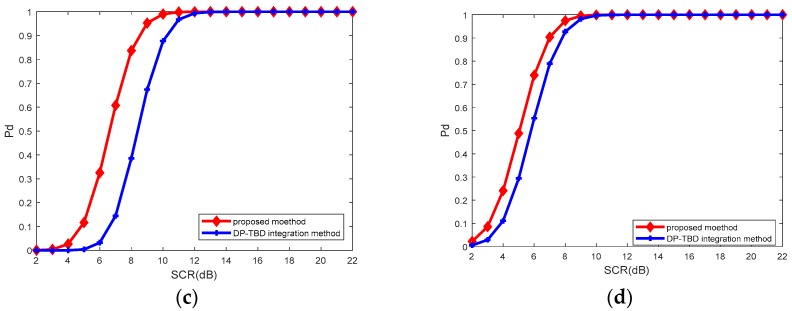
Performance comparison of DP–TBD integration method (red solid line with diamond) and the proposed method in this paper (blue solid line with cross) for K-distributed clutter and a Swerling 1 target (**a**) α=2 and β=2; (**b**) α=5 and β=2; (**c**) α=10 and β=2; (**d**) α=50 and β=2.

For different parameters of K-distributed clutter, detection performances are shown in Figure 7. With an increase of shape parameter α, both conventional DP and the proposed method on prior information achieve significant performance improvement. That is because when α is increasing, the K-distributed clutter is smoother and the frequency of target-like outliers is lower. Note that when α = 50, since K distribution almost degenerates to Rayleigh distribution in this case, the detection performances are nearly identical.

### 4.2. Computational Complexity Analysis

The complexity of the conventional DP–TBD method is $O\left(M_{r}M_{\theta }qN\right)$, where $M_{r}$ and $M_{\theta }$ are the number of range and azimuth resolution elements, *q* is the number of possible state transitions in a scan, and N is the number of the integration scans. In comparison with the conventional method, the two-stage detection approach schemed in Figure 3 has low computational complexity.

**Figure 8:**
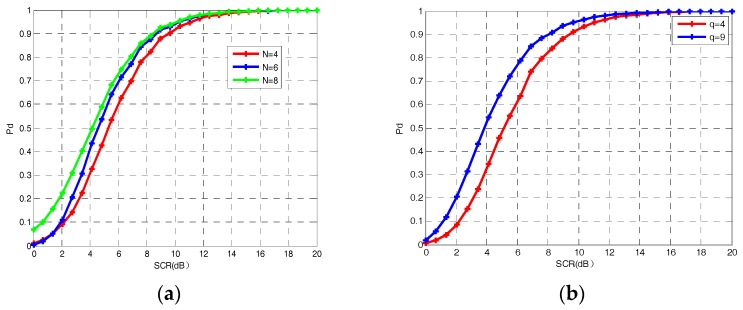
Performance comparison of DP–TBD integration method. (**a**) Performance with different number of frames *N* = 4, *N* = 6 and *N* = 8; (**b**) performance with different number of state transitions *q* = 4 and *q* = 9.

For a different number of frames and different number of state transitions, detection performances are shown in Figure 8. With the increase of frames and state transitions, the proposed method achieves a performance improvement, but not very much. However, the computational cost increases rapidly.

The computational cost in stage 1 is $O\left(M_{r}^{\prime }M_{\theta }^{\prime }qN\right)$, where $M_{r}^{\prime }\times M_{\theta }^{\prime }$ denotes grid cells re-discretized by $∆r^{\prime }$ and $∆\theta^{\prime }$ to realize the target search with larger cell size. Since the DP integration in stage 2 is concentrated on the part of states, which are indicated by stage 1, the computational cost is small enough not to care.

The computational cost of the strategies is listed in Table 2 for different parameters. It can be seen that the computational cost depends on the number of a possible state transition q and the resolution elements. For the same resolution cells, the scenario at *q* = 9 costs almost three times as long as the scenario *q* = 4. Meanwhile, the computational cost reduces rapidly as the number of resolution elements decreases. For example, when *q* = 4, the CPU times for $M_{r}\times M_{\theta }=180\times 90$, $M_{r}^{\prime }\times M_{\theta }^{\prime }=90\times 45$ and $M_{r}^{\prime }\times M_{\theta }^{\prime }=60\times 30$ are 308 ms, 224 ms and 146 ms, respectively.

## 5. Conclusions

This paper has presented the systematic treatment of heavy-tailed clutter from a target detection and tracking perspective. Target detection in K-distributed clutter is more challenging than in Gaussian- or Rayleigh-distributed clutter due to the higher likelihood of target-like outliers, especially for a fluctuating target. In this work, we dealt with the fluctuating target detection and tracking problem using a modified DP–TBD method. The contributions are as follows: first we have solved the target detection problem using two-stage detection architecture to avoid calculations of less meaningful states. Secondly, for a Swerling 1 target in a K-distributed background, the merit function was derived and implemented in the integration process of DP–TBD to enhance radar detection performance. In order to reduce the complexity of integral calculation, we also resorted to the trapezoidal rule with a generating lookup table.

Numerical analysis demonstrated that performance improvement could be applied via the proposed DP–TBD algorithm based on prior information, especially for heavy-tailed K-distributed clutter. Moreover, simulation results suggested that a trade-off between performance and computational complexity exists. Further research may investigate the performance of the proposed DP–TBD method experimentally. It may also be of interest to investigate other background models than the K-distribution.

## Figures and Tables

**Table 1:** Algorithmic description of the proposed two-stage TBD.

	Stage 1
Mearsurement:	get ${z_{n}}^{\prime }\left(i,j\right),1\leq i\leq M_{r}^{\prime },1\leq j\leq M_{\theta }^{\prime },for\;n=1,\ldots N$
Integration:	$V{\left(s_{n}\right)}^{\prime }=\sqrt{I{\left(s_{n}\right)}^{\prime }}+\underset{s_{n-1}\in \tau \left(s_{n-1}\right)}{\max}\left[V{\left(s_{n-1}\right)}^{\prime }\right],for\;n=1,\ldots N$ integration calculates under the condition of low grid resolution
Determination:	$V{\left(s_{N}\right)}^{\prime }>\gamma_{1}$
	Stage 2
Mearsurement:	get $z_{n}\left(i,j\right),1\leq i\leq M_{r},1\leq j\leq M_{\theta },for\;n=1,\ldots N$
Integration:	$V\left(s_{n}\right)=\sqrt{I\left(s_{n}\right)}+\underset{s_{n-1}\in \tau \left(s_{n-1}\right)}{\max}\left[V\left(s_{n-1}\right)\right],for\;n=1,\ldots N$ integration concentrates on the part of states which are indicated by stage 1
Determination:	$V\left(s_{N}\right)>\gamma_{2}$
Backtracking:	${\hat{S}}_{N}=\left\{{\hat{s}}_{1},\ldots ,{\hat{s}}_{N}\right\}=\arg\underset{s_{n-1}\in \tau \left(s_{n}\right)}{\max}\left[V\left(s_{n-1}\right)\right],for\;n=N,\ldots 1$

**Table 2:** Computational cost with different parameters.

Parameters	$M_{r}\times M_{\theta }=180\times 90$	$M_{r}^{\prime }\times M_{\theta }^{\prime }=90\times 45$	$M_{r}^{\prime }\times M_{\theta }^{\prime }=60\times 30$
*q* = 4, *N* = 6	308 ms	224 ms	146 ms
*q* = 9, *N* = 6	935 ms	684 ms	370 ms
